# Low-Cost Angle Sensor for Robotics Applications Using Plastic Optical Fiber Based on Optical Loss Mechanism

**DOI:** 10.3390/biomimetics8080567

**Published:** 2023-11-25

**Authors:** Hyun-Woo Lee, Dae-Hyun Kim, Sangwoo Shin

**Affiliations:** 1 Mechanical Engineering, Seoul National University of Science & Technology, 232 Gongneung-ro, Nowon-gu, Seoul 01811, Republic of Korea; leehyunwoo@seoultech.ac.kr; 2 Department of Mechanical & Automotive Engineering, Seoul National University of Science & Technology, 232 Gongneung-ro, Nowon-gu, Seoul 01811, Republic of Korea; 3 Department of Mechanical and Aerospace Engineering, University at Buffalo, The State University of New York, Buffalo, NY 14260, USA; sangwoos@buffalo.edu

**Keywords:** step index profile plastic optical fiber (SI-POF), bending loss, coupling loss, robot arm joint, underwater, electromagnetic interference (EMI)

## Abstract

Robotic systems and the human body consist of numerous joint structures, all of which require precise angle adjustments. At present, encoder, strain gauge, and electrical resistance-based sensors are commonly used for angle measurement. However, these sensors have limitations when used in underwater or in environments with strong electromagnetic waves. Therefore, we have developed an angle sensor based on step-index profile plastic optical fiber (SI-POF), which is cost-effective and highly durable, in this study in order to overcome the limitations of existing angle measurement sensors. To this end, the amount of light loss according to the gab and angle changes that occur when the POF angle sensor is applied to the robot arm was experimentally measured, and based on the results, a simulation of the amount of light loss when the two losses occurred at the same time was conducted. In addition, the performance of the POF angle sensor was evaluated by measuring sensitivity and resolution, and comparative verification with a commonly used encoder was conducted to verify the reliability of sensors in extreme environments, such as those with electromagnetic fields and those that are underwater. Through this, the reliability and practicality of the POF angle sensor were confirmed. The results obtained in this study suggest that POF-based angle sensors can contribute to the development of the biomimetic robot industry as well as ordinary robots, especially in environments where existing sensors are difficult to apply, such as areas with underwater or electromagnetic interference (EMI).

## 1. Introduction

Angle measurement is used in a variety of industries, from the traditional mechanical industry to clinical and motion capture, and related research is actively underway [1,2,3,4]. In particular, various robot systems including biomimetics, the use of which has recently been rapidly increasing, are composed of many joint structures, and the joint structures necessarily involve angle adjustment. An encoder, strain gauge, and electric resistance-based sensor are commonly used as conventional angle measurement methods [3,4,5]. However, these sensors have limitations in their use in extreme environments, such as those with strong electromagnetic waves or that are underwater, acting as an obstacle to expanding the application area of robotic systems. In contrast, optical fiber sensors have no effect on electromagnetic waves, have a wide operating temperature range, and are free from the effects of water or corrosion. In addition, it has many advantages, such as being able to transmit signals over long distances with little loss and being easy to embed due to its small size.

Fiber optic sensors can be classified by light intensity type [6,7], interference type [8], scattering type [9], and wavelength shift type [10,11,12,13,14,15,16] depending on the demodulation method. Among them, a wavelength shift type Fiber Bragg grating (FBG) sensor has been studied in depth for angle and shape estimation [15,16], but the optical system for FBG operation is very expensive, about USD 20,000 or more, which limits its application. In contrast, light intensity fiber optic sensors with easy demodulation techniques and low system prices are expanding their application areas in various industries. In particular, plastic optical fiber (POF), which is relatively durable compared to silica-based optical fiber, is highly applicable to the composition of light intensity sensors, and the system configuration price is as low as USD 30 or less [17,18]. In addition, POF is easy to handle because it has a large core diameter and numerical aperture (N.A.) [18], and has excellent flexibility, so related research has been expanding recently. Therefore, it can be a good option as a sensor for measuring angles when applied to joint parts such as robot arms in an extreme environment.

**Figure 1:**
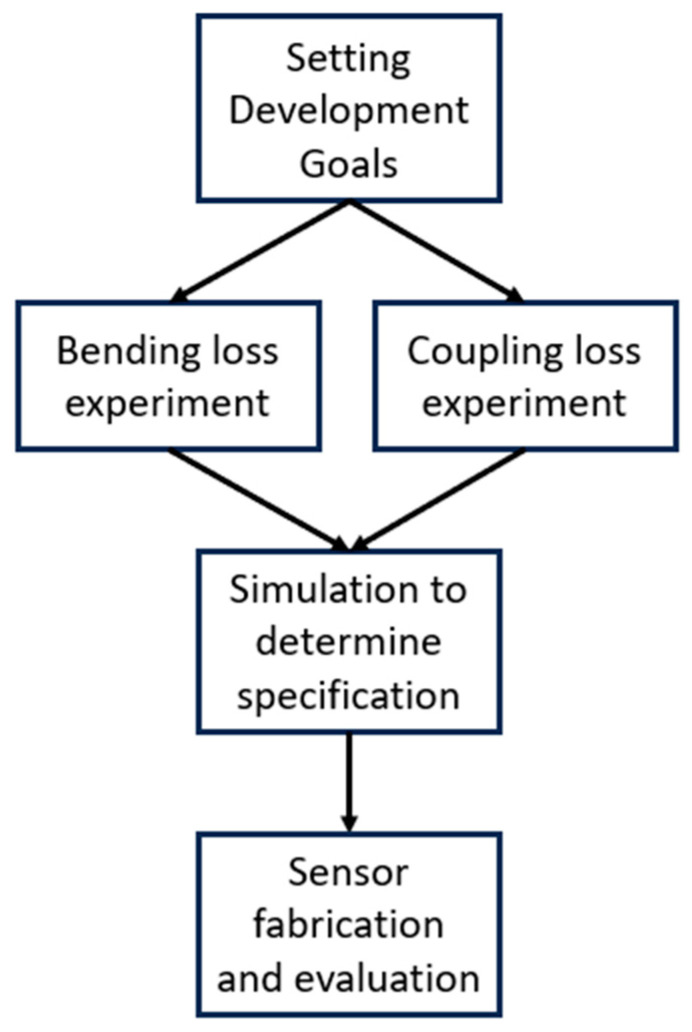
Research flow chart.

Therefore, in this study, we developed a cost-effective and easy-to-handle POF angle sensor to overcome the limitations of existing angle sensors. The process is illustrated in the flowchart presented in Figure 1. First, as described above, the development goal was set to create a POF angle sensor that can operate stably in an extreme environment. Second, for POF angle sensor development, the amount of light loss caused by the gab change and angle change generated when a POF angle sensor was applied to the robot arm was experimentally measured. Third, based on the previous results, a simulation of the amount of light loss when the two losses occurred at the same time was conducted. Fourth, the POF angle sensor proposed in this paper was applied to the robot arm joint structure and the performance of the sensor was verified by measuring sensitivity and resolution according to the change in the robot arm rotation angle. Also, comparative verification with a commonly used encoder was conducted to verify the reliability of the POF angle sensor proposed in this paper in extreme environments such as those with electromagnetic fields and that are underwater. Through this, the reliability and practicality of POF angle sensors were evaluated.

## 2. Light Loss Theory of POF

### 2.1. Bending Loss of POF

**Figure 2:**
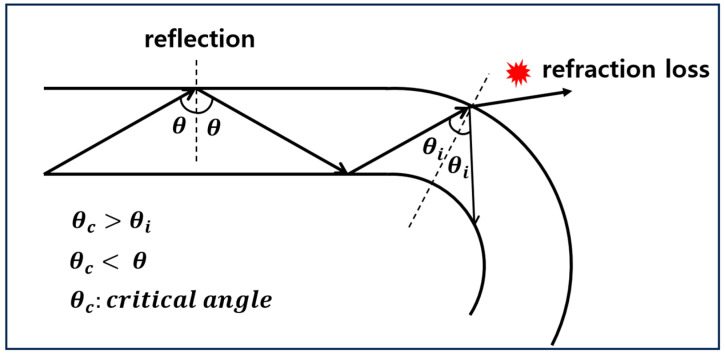
Refractive loss due to reduced angle of incidence.

The theoretical analysis of the bending loss of multi-mode step-index profile plastic optical fiber (SI-POF) is based on geometric optical analysis. The light ray incident on the optical fiber is divided into a meridional ray that passes through the central axis of the core and a skew ray that does not pass through the central axis of the core. Most of the losses that occur when bending is applied to an optical fiber occur in meridional rays, so losses in skew rays are not considered [19]. When meridional rays traveling inside an optical fiber are incident on the interface between the core and cladding, two types of optical loss occur. The first is when the incident angle ($\theta_{i}$) is smaller than the critical angle ($\theta_{c}$). In this case, the light ray is refracted at the boundary and loss occurs. The second is when the incident angle ($\theta_{i}$) is equal to or greater than the critical angle ($\theta_{c}$), and the light ray leaks from the boundary and a relatively small loss occurs [19,20]. When curvature is applied to the optical fiber, the angle of incidence becomes smaller, as shown in Figure 2. Accordingly, through this principle, light loss due to refraction occurs in light rays whose incidence angle is smaller than the critical angle, which is expressed in Equation (1).

(1) $$P_{r}=P_{i}\left(1-T\right)$$

Here,$\;P_{i}$ and $P_{r}$ are the incident and reflected rays, respectively, and T is the power transmission coefficient, calculated using Equation (2) [21].

(2) $$T=\frac{4\cos\theta_{i}{\left(\cos^{2}\theta_{i}-\cos^{2}\theta_{c}\right)}^{1/2}}{{\left[\cos\theta_{i}+{\left(\cos^{2}\theta_{i}-\cos^{2}\theta_{c}\right)}^{1/2}\right]}^{2}}$$

In this way, the light loss resulting from curvature can be calculated through the sum of light losses due to refraction from all rays incident at an angle (−$\theta_{c}<\theta <+\theta_{c}$) smaller than the critical angle ($\theta_{c}$) [19].

**Figure 3:**
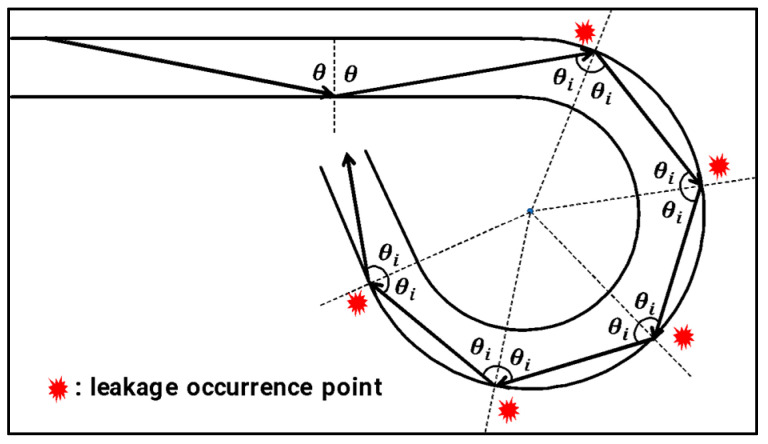
A beam of light continuing at the same angle of incidence within an arc.

On the other hand, light rays with an incident angle ($\theta_{i}$) greater than the critical angle ($\theta_{c}$) continue to be reflected at the same incident angle within a curvature with a constant radius of curvature, as shown in Figure 3, and proceed along the curvature, and loss due to continuous leakage occurs in this process.

However, in the case of previous research [19], only the bending loss of optical fibers due to refraction, which occurs mostly at the beginning of curvature, is considered. On the other hand, cases where continuous loss due to leakage occurs, as shown in Figure 3, are not considered, so there is a limit to its application. In this paper, therefore, we overcome the limitations of existing theoretical analysis and experimentally measured the optical loss that occurs when optical fibers are used as angle sensors.

### 2.2. Coupling Loss of POF

**Figure 4:**
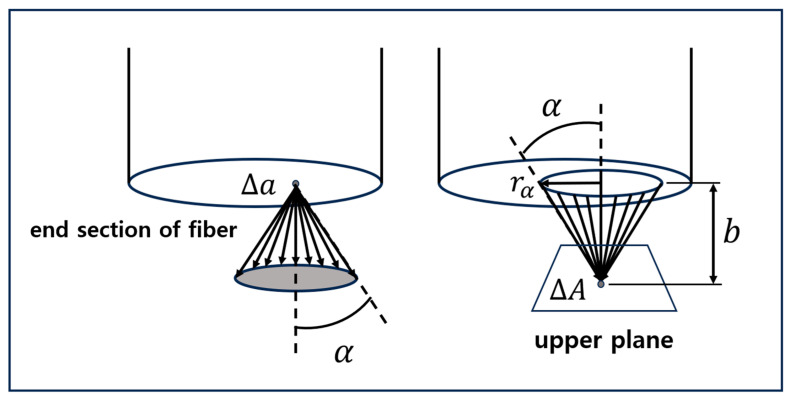
Basic concepts of light intensity distribution modeling [22].

**Figure 5:**
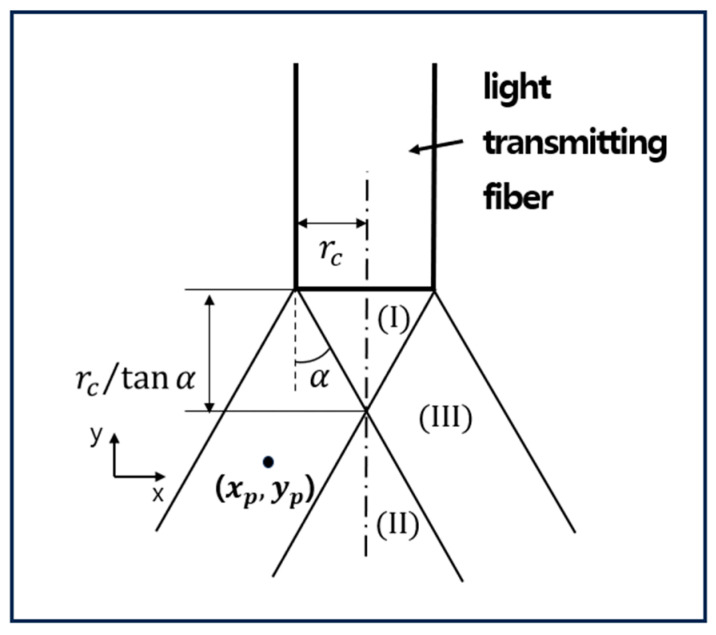
Illuminance distribution by plastic optical fiber area [22].

The coupling loss according to the gap distance between two aligned plastic optical fibers can be calculated through the illuminance distribution model of optical fibers based on photometric analysis [22]. In other words, in a light-transmitting fiber optical, as shown in Figure 4, the radiance of a small area Δα on the end surface and the size of the irradiance of a small area ΔA of the image plane that receives the light are shown for each position. Through Figure 5, the amount of the irradiance distribution for each area can be obtained, and the amount of the irradiance varies depending on the irradiation location $\left(x_{p},y_{p}\right)$. In Figure 5, the irradiance equations for areas (I), (II), and (III) can be obtained through Equations (3), (4), and (5), respectively.

(3) $$E_{I}=M$$

(4) $$E_{\mathrm{II}}=\frac{L\pi }{2}\left[1-\cos\left(\theta_{2}-\theta_{1}\right)\right]$$

(5) $$E_{\mathrm{III}}=2\left[\begin{array}{c} \int_{x_{p}-r_{\alpha }}^{x_{0}}\int_{0}^{\sqrt{r_{\alpha }^{2}-{\left(x-x_{p}\right)}^{2}}}\frac{Ly_{p}^{2}}{{\left(y_{p}^{2}+z^{2}+{\left(x_{p}-x\right)}^{2}\right)}^{2}}dzdx\\ +\int_{x_{0}}^{r_{c}}\int_{0}^{\sqrt{r_{c}^{2}-x^{2}}}\frac{Ly_{p}^{2}}{{\left(y_{p}^{2}+z^{2}+{\left(x_{p}-x\right)}^{2}\right)}^{2}}dzdx \end{array}\right]$$

Here, L is the radiant luminance of a small area and M is the radiative divergence of the light-transmitting fiber, which has the relationship shown in Equation (6).

(6) $$M=\frac{\pi L}{2}\left(1-\cos2\alpha \right)$$

Additionally, $\theta_{1}$ and $\theta_{2}$ in Equation (4) are calculated through Equations (7) and (8), and $r_{\alpha }$ and $x_{0}$ in Equation (5) are calculated through Equations (9) and (10), respectively.

(7) $$\theta_{1}=\sin^{-1}\frac{r_{c}-x_{p}}{\sqrt{y_{p}^{2}+{\left(r_{c}-x_{p}\right)}^{2}}}$$

(8) $$\theta_{2}=\sin^{-1}\frac{r_{c}-x_{p}}{\sqrt{y_{p}^{2}+{\left(r_{c}+x_{p}\right)}^{2}}}$$

(9) $$r_{\alpha }=\sqrt{{\left(x-x_{p}\right)}^{2}+z^{2}}$$

(10) $$x_{0}=\frac{r_{c}^{2}-r_{\alpha }^{2}+x_{p}^{2}}{2x_{p}}$$

## 3. Experiment for Optical Loss of POF

### 3.1. Experimental Apparatus and Method

In this section, a quantitative evaluation was conducted using experimental methods for the two types of light loss.

**Figure 6:**
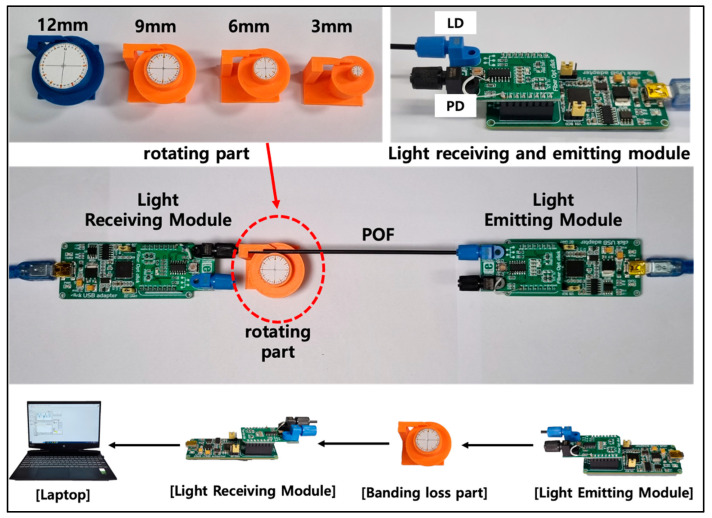
Experimental setup for a bending loss test.

First, an experimental device, as shown in Figure 6, was prepared to measure bending loss as the rotation angle increases when the robot arm rotates. In addition, considering rotating parts with various radii, test specimens simulating a rotating part with four types of radii, including 3 mm, 6 mm, 9 mm, and 12 mm, were produced using a 3D printer.

The experiment was conducted by measuring the amount of light loss that occurs when an optical fiber is wound around each manufactured rotating part at 30-degree intervals from 0 degrees to 360 degrees. At this time, a constant load was applied to the optical fiber to ensure that there was no problem with the optical fiber’s followability when the radius of curvature changed.

**Figure 7:**
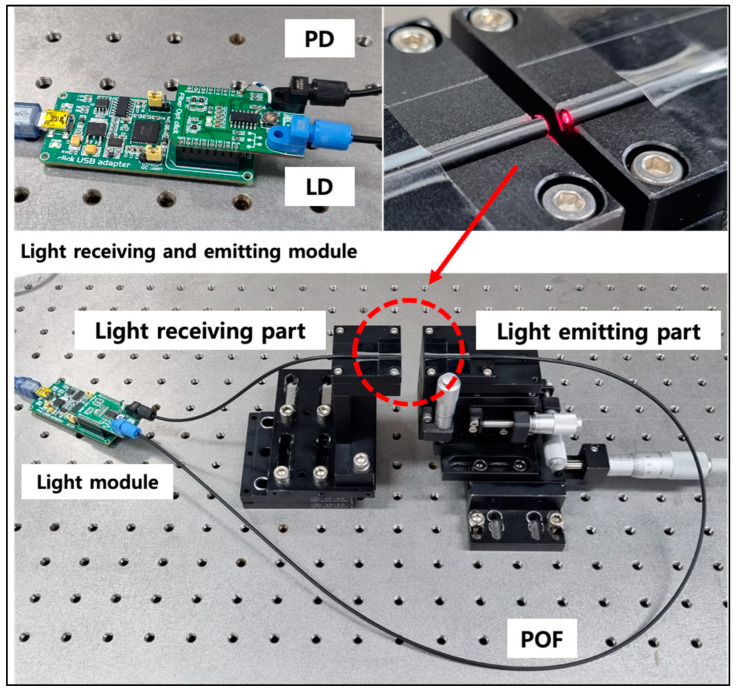
Experimental setup for a coupling loss test.

Second, an experimental device, as shown in Figure 7, was prepared to measure coupling loss according to the change in gap distance between two optical fibers. The experiment was conducted by aligning two strands of optical fibers on the same line using a 3-axis moving stage equipped with a micrometer and then changing the gap distance between the optical fibers. The amount of light loss occurring when the gap distance was changed from 0.0 to 39.0 mm at 1.0 mm intervals was measured.

In both types of experiments, GH4001, a multimode SI-POF from Eska™, was used as the optical fiber. The numerical aperture (N.A.) of the optical fiber used was 0.5, and the diameters of the core, cladding, and jacket were 980 μm, 1000 μm, and 2.2 mm, respectively, and the materials were polymethyl methacrylate, fluorinated polymer, and polyethylene, respectively. LD (IFE97, Industrial Fiber Optics) with a wavelength of approximately 660 nm was used as the light emitting module. In addition, as a light receiving module, a Fiber Opt click (Mikroe-1940, Mikro Elektronika d.o.o, Belgrade, Serbia) with a built-in photodetector (IF D91B, Industrial Fiber Optics) of the same wavelength was used.

Additionally, the light intensity data were connected to the measurement computer through the same company’s click USB adapter (Mikroe-1433) and acquired by reading the output voltage through the LabVIEW software. All experiments were performed five times, and the measured values were averaged.

### 3.2. Experimental Results

**Figure 8:**
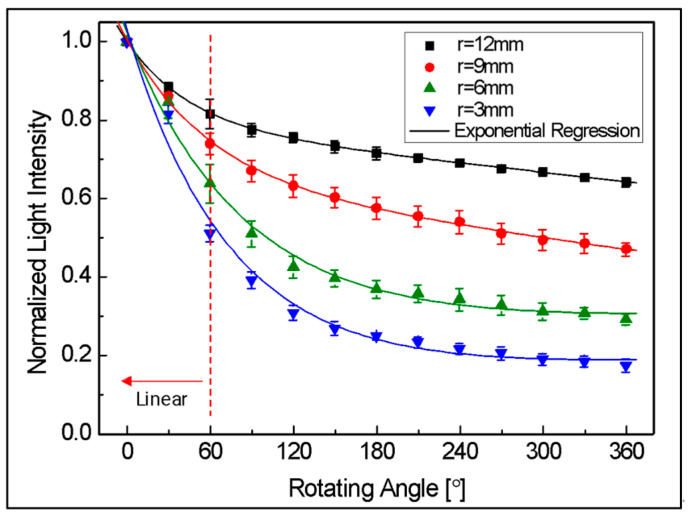
Normalized light intensity according to radius.

First, the results of the bending loss measurement experiment according to the change in the angle of the rotating part are shown in Figure 8. The changes in result values according to four types of changes in the radius of the rotating part are also presented. In the figure, as the rotation angle increases from 0 degrees to 360 degrees, it shows an almost linear decrease until about 60 degrees, and then the nonlinearity rapidly increases. However, in the section after 150 degrees, there is a characteristic that shows linearity again.

**Figure 9:**
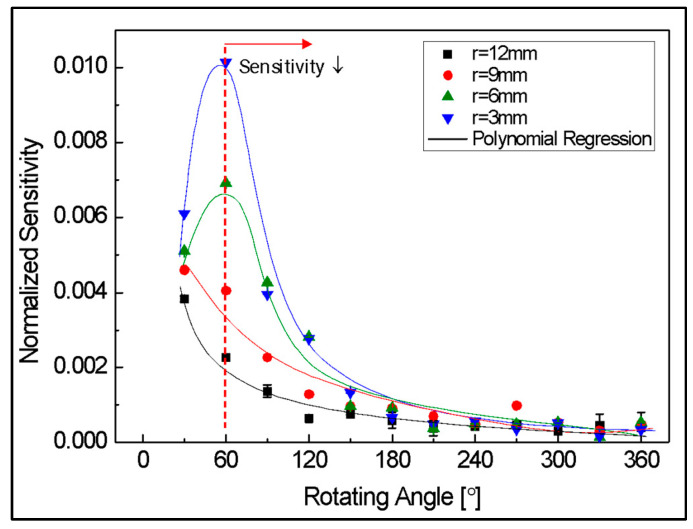
Normalized sensitivity of light intensity according to radius.

The sensitivity of banding loss is expressed in Figure 9. This indicated the average amount of light loss as a ratio of the initial amount of light as the angle changes by 1 degree within the 30-degree interval, and this result was approximated through exponential regression. In the range of 0 to 60 degrees, where linearity was good, sensitivity was also relatively excellent. From the geometrical optical theory in Section 2.1, it can be assumed that this is caused by loss due to refraction caused by a smaller angle of incidence at the beginning of the curvature. In addition, with respect to changes in the radius of the rotating part, if the radius is small, greater loss occurs depending on the angle change, so the sensor showed better results in terms of sensitivity. On the other hand, in the section after 60 degrees where nonlinearity increases, sensitivity is also gradually decreased. In particular, after about 150 degrees, the sensitivity shows a very low characteristic regardless of the change in the radius of the rotating part. This is because the influence of refraction loss is reduced in that section, and the influence of leakage loss occurring as it continues inside the curvature dominates. Therefore, as mentioned in a previous study [21], it was experimentally confirmed that at sufficient curvature, most of the refractive loss due to bending occurs in the section below about 120 degrees, and that leakage loss dominates in the section above 150 degrees.

**Figure 10:**
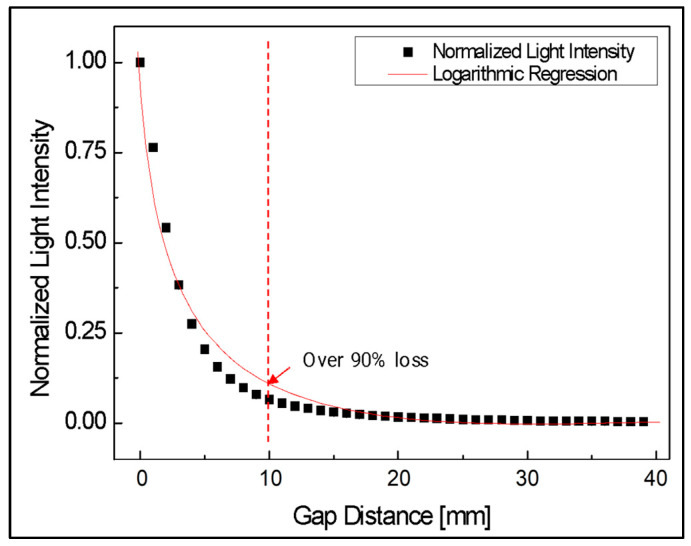
Normalized light intensity according to gap between two aligned optical fibers.

Second, the results of the coupling loss experiment according to the change in the gap between the two optical fibers are shown in Figure 10. Overall, the larger the gap, the greater the light loss. In particular, the degree of change rapidly decreased logarithmically as the gap increased. In addition, a high light loss of more than 90% occurred at a 10 mm gap distance.

**Figure 11:**
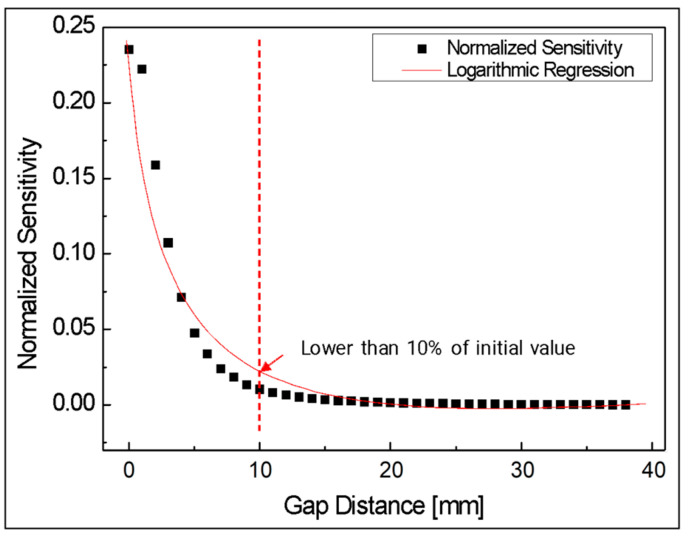
Normalized sensitivity of light intensity according to gap between two aligned optical fibers.

The sensitivity of coupling loss is expressed in Figure 11. This indicated the amount of light loss as a ratio of the initial amount of light as the gap changed by 1 mm, and this result was approximated through logarithmic regression. It showed a similar tendency to the results of Figure 10. The sensitivity at a gap distance of 10 mm was very low at less than 10% of the initial sensitivity.

## 4. Robot Arm Device with POF Angle Sensor

### 4.1. POF Applied Robot Arm Device Design

In this chapter, a POF angle sensor applicable to a robot arm was manufactured using bending loss and coupling loss of POF, which were experimentally confirmed in Section 3.

**Figure 12:**
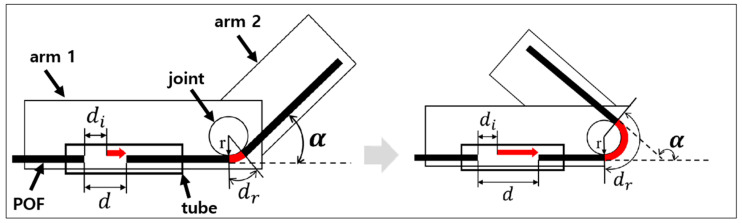
Conceptual approach for rotating angle detection mechanism.

First, the internal structure of the manufactured POF angle sensor was designed as shown in Figure 12. For easy application to robots, the POF angle sensor was designed based on the robot’s arm and joint structure. The sensor is a form in which two arms are joined by a joint and can freely adjust the angle (α). Inside the tube installed on arm 1, two optical fibers are aligned in a straight line and designed to face each other. Based on the tube, the optical fiber on the left is fixed, and the optical fiber on the right is guided to pass through the curvature created in the joint as the device is given an angle, resulting in a distance from the left optical fiber inside the tube. As a result, as the gap between optical fibers inside the tube widens, light flows out, causing coupling loss, and the right optical fiber also causes bending loss due to curvature. Through this, a structure is formed in which bending loss and coupling loss occur simultaneously in the POF angle sensor when the angle of the robot arm rotating part changes.

Here, the gap distance (d) between two optical fibers is the sum of the length ($d_{r}$) of the optical fiber surrounding the curvature created by the angle (α) and the initial gap ($d_{i}$) between the two optical fibers, which is equal to Equation (11). In addition, the length ($d_{r}$) of the optical fiber surrounding the curvature of the rotating part is calculated as in Equation (12), considering the radius of the POF of 1.1 mm.

(11) $$d=d_{i}+d_{r}$$

(12) $$d_{r}=\frac{\alpha }{360}\times 2\pi \left(r+1.1\right)$$

Meanwhile, when bending loss and coupling loss act in combination, the light quantity (P) at each angle can be expressed as the product of the light quantity ($P_{b}$) when bending loss occurs and the light quantity ($P_{c}$) when coupling loss occurs, as shown in Equation (13).

(13) $$P=P_{b}\times P_{c}$$

To determine the specifications of the joint to be applied to the robot arm, a simulation was conducted to determine the initial gap distance ($d_{i}$) between the two optical fibers and the radius of the joint (r). The values of Figure 8 and Figure 10 obtained through the experiments in Section 3 were substituted into Equation (13) to analyze the characteristics according to the initial gap and the radius of rotation of the joint.

**Figure 13:**
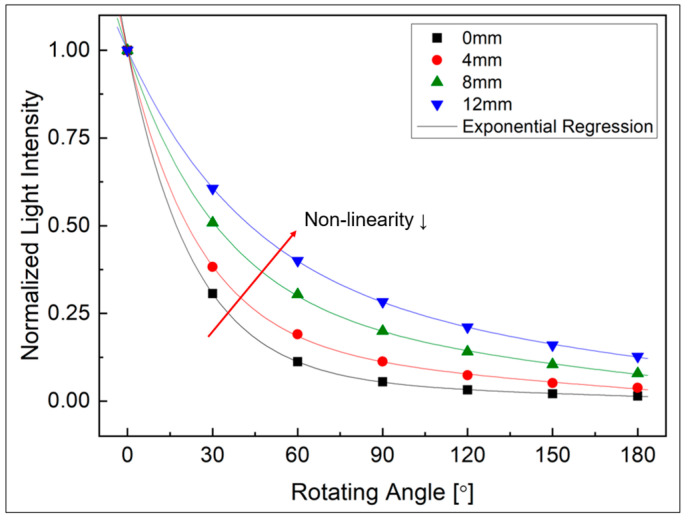
Normalized light intensity with initial gap of 0 mm, 4 mm, 8 mm and 12 mm (at r = 6 mm).

First, to determine the initial gap distance, the joint radius was fixed to 6 mm among the four types used in this study, and the change in light intensity according to the gap distance change was calculated. The initial gap distance was changed from 0 to 12 mm at 4 mm intervals, and the change in total light intensity was expressed as a ratio the initial value, as shown in Figure 13. In the figure, as the initial gap distance increases, the nonlinearity gradually decreases, which can be understood as the reason why the change in sensitivity is large at a gap distance of less than 10 mm in the results in Figure 11. In this study, therefore, the initial gap distance was decided to be 8 mm by comprehensively considering the decrease in the amount of light due to the increase in gap distance and the linearity issue required for the sensor.

**Figure 14:**
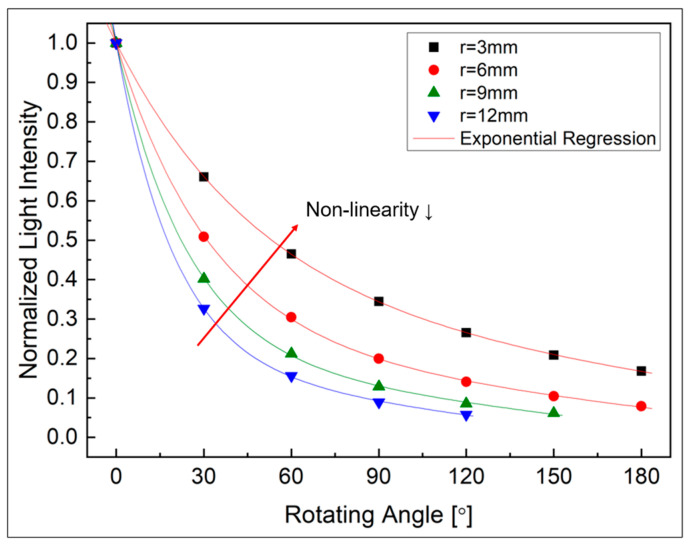
Normalized light intensity by radius change in rotating part (at $d_{i}$ = 8 mm).

Second, to determine the radius of the joint, the gap distance was fixed to the previously determined 8 mm, and the change in light intensity was calculated according to the change in radius. The change in total light intensity according to the angle change for four types of radii of 3, 6, 9, and 12 mm was expressed as a ratio of the initial value, as shown in Figure 14. From the figure, it can be seen that as the radius becomes smaller, the amount of light changes linearly with respect to the angle change. This can be understood because the smaller the radius, the smaller the absolute movement amount due to rotation ($d_{r}$), and the increase in nonlinearity in Equation (13) also decreases. However, in order to manufacture a device, a radius of a certain size or more is required and the bending limit of the optical fiber itself (possibility of birefringence, etc.) need to be taken into consideration. Therefore, considering these points comprehensively, the minimum radius that can be manufactured was determined to be 7.5 mm.

**Figure 15:**
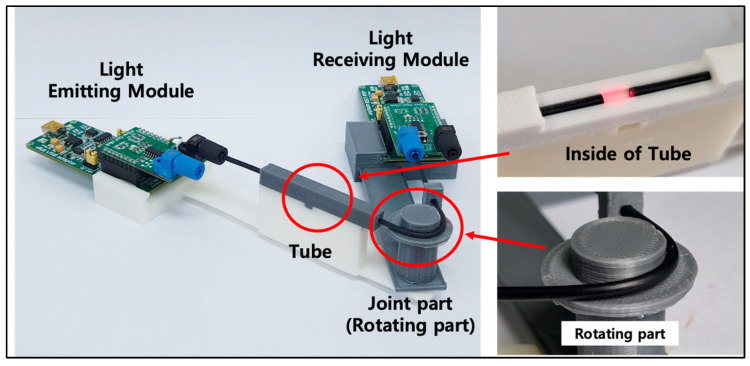
A robot arm part device with POF angle sensor.

Based on the above results, a robot arm joint structure, as shown in Figure 15, was manufactured. In Figure 15, the device consists of a light receiver module, a light emitter module, a tube, and a joint, and each component was manufactured using a 3D printer. The joint is a structure in which two frames are joined by pins, allowing for free angle adjustment.

### 4.2. Performance Evaluation of POF Angle Sensor

**Figure 16:**
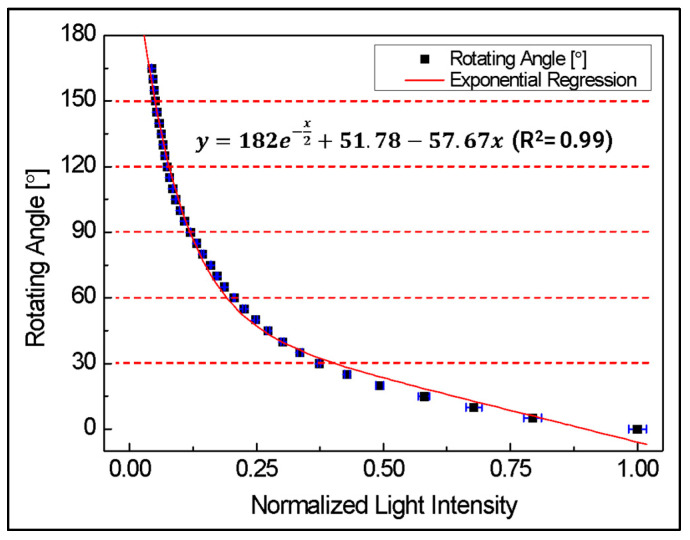
Performance test result of POF angle sensor.

Figure 16 shows the results of an experiment measuring light intensity at each angle, repeated 5 times at 5-degree intervals from 0 to 165 degrees using the manufactured POF angle sensor. From the experimental results, the relationship between the change in rotation angle and the change in total light intensity can be derived as Equation (14).

(14) $$y=182\;e^{-\frac{x}{2}}+51.78-57.67\;x$$

Here, x is the amount of optical loss normalized to the initial amount of optical loss, and y is the rotation angle of the joint part.

From Equation (14), the rotation angle can be calculated by measuring the amount of light loss, but it can be seen that the overall nonlinearity is large. However, when divided into 30-degree sections, a linearity of at least R^2^ = 0.95 or higher can be confirmed, as shown in Table 1. Therefore, by using this, it is possible to sufficiently confirm its applicability as an angle sensor.

**Figure 17:**
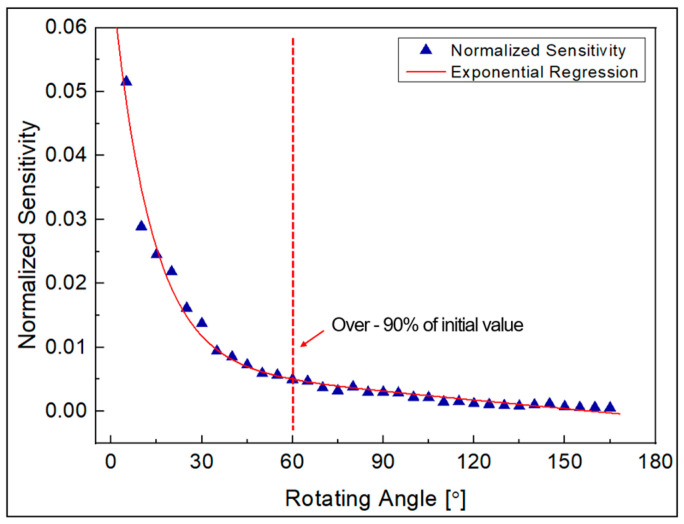
Normalized sensitivity of light intensity of POF angle sensor.

Meanwhile, Figure 17 shows the sensitivity to light measurement results by angle. This indicated the average amount of light loss as a ratio of the initial amount of light, as the angle changes by 1 degree within the 5-degree interval, and this result was approximated through exponential regression. In the figure, you can see that when the angle is small, the sensitivity is high, but at 60 degrees, the sensitivity decreases by more than 90% compared to the initial value. Additionally, in the section after 60 degrees, the change in light quantity can be assumed to be linear with respect to the angle change, so if it is used as a sensor using only that section, its usability as a sensor is good enough. However, in order to implement this, the optical fiber needs to be fixed for the initial 60-degree rotation amount, which requires additional work, such as the consideration of the fact that the operable angle range is limited and changes to the sensor structure.

In addition, the noise for each section was measured, and the resolution was obtained through this and displayed in Table 2. The resolution is a value obtained by dividing noise [mV] by the average sensitivity [mV/°] of each section, and the unit is a degree [°]. As the sensitivity decreases toward the rear section, the resolution tends to increase. Here, the noise was calculated as the width between the maximum and minimum values measured for 2 s at a sampling frequency of 50 Hz. It can be seen that noise exists almost uniformly regardless of the section.

### 4.3. Comparative Evaluation of Sensors in Extreme Environments

**Figure 18:**
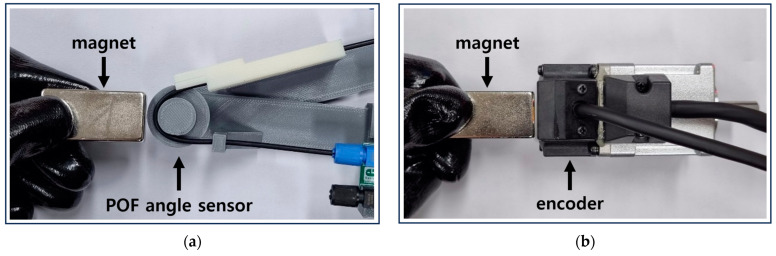
Exposing a magnetic field to each sensor: (**a**) POF angle sensor; (**b**) encoder.

In an additional experiment, the EMI influence of the POF angle sensor was compared with the encoder, a general angle sensor, to show the reliability of the POF angle sensor proposed in this study in an extreme environment. As shown in Figure 18, in order to artificially create an environment where a magnetic field exists, the experiment was conducted by repeatedly bringing the neodymium magnet with a maximum magnetic flux density of 0.412 Tesla close to the sensor within 10 mm and then placing it 150 mm away. Here, each sensor is fixed, and the magnet is prevented from touching the sensor. The encoder used in the experiment is the MSMF5AZL1S2 model from Panasonic Industry Co., Ltd. (Osaka, Japan).

**Figure 19:**
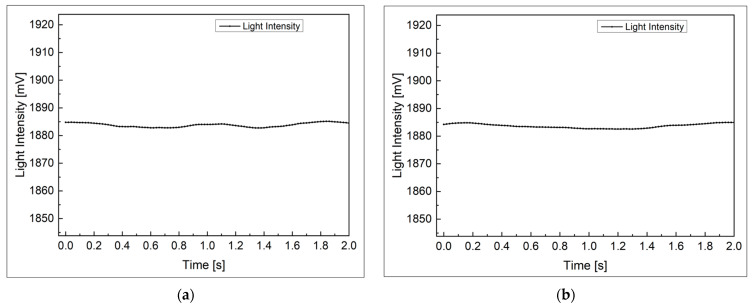
Signal of POF angle sensor: (**a**) noise in a general environment; (**b**) interference in the magnetic field environment.

**Figure 20:**
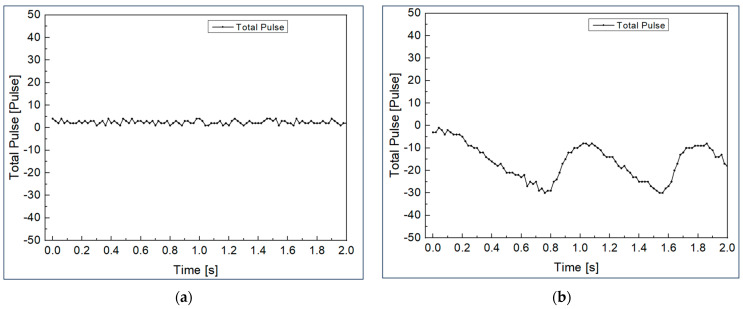
Signal of encoder: (**a**) noise in a general environment; (**b**) interference in the magnetic field environment.

For the POF angle sensor and the encoder, the noise in general environments and interference in environments exposed to magnetic fields are shown in the graphs of Figure 19 and Figure 20, respectively. In addition, the relative ratio of the interference level in the environment exposed to the magnetic field and the noise level in the general environment for each sensor is shown in Table 3. Here, the noise signal and the interference were measured for 2 s at a sampling frequency of 50 Hz, and the noise level and interference level were calculated as the width between the maximum and minimum of the measured signal. In the case of POF angle sensor, there is no meaningful difference in interference level, even when exposed to magnetic fields, but in the case of the encoder, the interference level was increased by 9.67 times compared to the noise level. This result is predicted to be much greater if the magnet is brought closer to the encoder, or if it exposed to a stronger magnetic field. In addition, encoders cannot be used in water unless they are waterproofed, but POF angle sensors can be used regardless of whether they are waterproof or not. The above results confirm that the POF angle sensors proposed in this paper are reliable in magnetic fields and underwater environments, unlike existing sensors. The results obtained in this study suggest that POF angle sensors can be useful in environments where existing sensors are difficult to apply, such as areas where electromagnetic interference (EMI) occurs or underwater, which can contribute to expanding the application area of robot systems.

## 5. Conclusions

In this study, we developed a POF angle sensor applicable to robot arms by utilizing the optical loss characteristics of POF, which is inexpensive and highly durable. The results obtained through this study are as follows.

(1) The characteristics of bending loss and coupling loss that occur when POF is applied to the joints of the robot arm were confirmed experimentally, and suitability was confirmed through comparison with theoretical equations from previous studies.
(2) A POF angle sensor capable of measuring robotic joint angles using both bending and coupling losses has been successfully designed and fabricated.
(3) When manufacturing the POF angle sensor for a robot arm using POF, the appropriate initial gap distance ($d_{i}$) and joint radius (r) were determined to be 8 mm and 7.5 mm, respectively, taking comprehensive consideration of manufacturability and optical fiber characteristics.
(4) Through performance evaluation of the manufactured POF angle sensor, it was confirmed that angle measurement up to 165 degrees is possible. In addition, it was confirmed that the POF angle sensor has linearity in each section and a minimum resolution of 0.030 degree in the 0–30 degree section. In addition, a method of limiting the initial angle of the sensor to 60 degrees was also proposed to improve the linearity of the sensor.
(5) In order to confirm the reliability of each sensor in an extreme environment, an interference measurement experiment was conducted in an environment with a maximum magnetic flux density of 0.412 Tesla. The encoder confirmed that the interference level in the magnetic field environment increased 9.67 times compared to the noise in the general environment, whereas it was confirmed that there was no difference in the POF angle sensor.

Furthermore, the POF angle sensor developed in this study is expected to contribute to the development of the biomimetic robot industry as well as ordinary robots, especially in environments where existing electrical sensors are difficult to apply, such as areas with underwater or electromagnetic interference (EMI). In addition, this paper proposed a new sensor that can secure usability even in an environment where existing sensors are difficult to apply, but there are still limitations in linear section expansion and sensor resolution improvement. Therefore, it is judged that related improvement research is necessary in the future.

## Figures and Tables

**Table 1:** R square value for each 30-degree section in Figure 16.

Section	0~30°	30~60°	60~90°	90~120°	120~150°	150~165°
$R^{2}$	0.95	0.98	0.99	0.97	0.99	0.99

**Table 2:** Resolution and noise value for each 30-degree section.

Section	0~30°	30~60°	60~90°	90~120°	120~150°	150~165°
Noise [mV]	2.39	2.324	2.375	2.443	2.405	2.442
Resolution [°]	0.030	0.109	0.218	0.418	0.816	1.333

**Table 3:** The relative ratio of the interference level in the environment exposed to the magnetic field, the noise level in the general environment, and usability in an underwater environment.

	Noise Level in a General Environment	Interference Level in Magnetic Field Environment	Relative Ratio of the Levels of Two Environments	Underwater Operation
Encoder	3 [Pulse]	29 [Pulse]	9.67	Inoperable
POF angle sensor	2.39 [mV]	2.40 [mV]	1.00	Operable

## Data Availability

Data are included within this article.
